# A Facile Profiling Method of Short Chain Fatty Acids Using Liquid Chromatography-Mass Spectrometry

**DOI:** 10.3390/metabo9090173

**Published:** 2019-08-28

**Authors:** Ha Eun Song, Hyo Yeong Lee, Su Jung Kim, Sung Hoon Back, Hyun Ju Yoo

**Affiliations:** 1 Department of Convergence Medicine, Asan Institute for Life Sciences, Asan Medical Center, University of Ulsan College of Medicine, Seoul 05505, Korea; 2 School of Biological Sciences, University of Ulsan, Ulsan 44610, Korea

**Keywords:** short chain fatty acids, 4-acetamido-7-mercapto-2,1,3-benzoxadiazole, chemical derivatization, exhaled breath condensate, feces, plasma

## Abstract

Short chain fatty acids (SCFAs) are the main products of dietary fibers that are not digested by the human body, and they have been shown to affect human metabolism and inflammation. The amount of SCFAs in the body is related to many human diseases, and studies have focused on elucidating their roles and target molecules in both metabolic and immune responses. Thus, the quantitation of SCFAs in biological samples becomes crucial in understanding their important roles in the human body. Herein, a facile profiling method of SCFAs using liquid chromatography-tandem mass spectrometry (LC-MS/MS) was developed and then applied to biological samples. C2-C6 SCFAs were derivatized while using 4-acetamido-7-mercapto-2,1,3-benzoxadiazole for 5 min. at room temperature prior to LC-MS/MS analysis, and characteristic fragmentation patterns and increased hydrophobicity after chemical derivatization enabled specific discrimination among 12 SCFAs. Derivatization was fast and reliable, and the reaction products were stable for a week at 4 °C. The developed method was applied to measure SCFAs in mouse feces, plasma, and human exhaled breath condensates. This fast and simple method can save labor and effort to profile SCFAs from various biological samples.

## 1. Introduction

Short chain fatty acids (SCFAs) contain less than six carbons, and they are mainly produced by the fermentation of dietary fibers in the human body [1]. One of the major roles of the gut microbiota is to help catabolize dietary fibers into SCFAs. SCFAs are taken up by the host and are used as energy sources or regulators [2]. The main SCFAs are acetate (C2), propionate (C3), and butyrate (C4), and they constitute 95% of total SCFAs. Straight chain SCFAs are derived from dietary fibers, while the branched chain SCFAs are derived from catabolism of branched chain amino acids [2]. SCFAs are metabolized at various sites in the body, transported from the intestinal lumen into the blood, and found in various tissues. Recent studies demonstrated that the gut microbiota plays an important role in regulating host metabolism and immune responses [3,4]. Thus, measuring the type and amount of SCFAs is important for understanding their roles in complex biological systems.

Mass spectrometry (MS) has grown in popularity as an analytical method for the determination of various biomolecules, and it is frequently used in combination with separation techniques, such as GC or HPLC, because biological samples are too complex for direct analysis by MS alone. SCFAs have been derivatized by methyl-, ethyl-, and propyl-chloroformate, as well as trimethylsilylation, and determined by GC-MS [5,6,7,8]. Liquid chromatography-mass spectrometry (LC-MS) has been often used in metabolomics studies with minimal sample preparation as compared with GC or GC-MS [9,10]. However, the quantitation of SCFAs without chemical derivatization requires harsh experimental conditions in LC-MS, such as an aqueous mobile phase containing 1.5 mM hydrochloric acid [11]. In addition, their hydrophilicity results in poor chromatographic separation and insufficient ionization in electrospray ionization (ESI) [11]. Thus, it was difficult to detect SCFAs by LC-MS, because their masses were in the lower mass range in mass spectra, where numerous interfering peaks from solvents and additives were present. To overcome these problems, several chemical derivatization methods have been introduced to quantify SCFAs while using LC-MS. However, these derivatization requires longer reaction time or specific reaction condition. SCFAs were derivatized with ^12^C- or ^13^C-labeled aniline and analyzed while using a reversed-phase LC column, where derivatization was performed for 2 h at 4 °C, and quenching was necessary to avoid unintended reactions [12]. In other studies, optimal reaction condition for derivatization of SCFAs with 3-nitrophenylhydrazine and O-benzylhydroxylamine was determined in 30 min. at 40 °C and 1 h at 25 °C, respectively [13,14]. 

In the present study, we aimed to develop a simple profiling method of SCFAs from various biological samples. SCFAs were derivatized with 4-acetoamido-7-mercapto-2,1,3-benzoxadiazole (AABD-SH) for 5 min. at room temperature. Derivatization reaction condition was optimized, and the performance of this method was evaluated with standard solutions and biological samples.

## 2. Materials and Methods 

### 2.1. Materials

Acetic acid (C2), propionic acid (C3), butyric acid(C4), isobutyric acid (C4; 2-methylpropionic acid), 2-methylbutyric acid (C5), isovaleric acid (C5; 3-methylbutyric acid), valeric acid (C5; pentanoic acid), 2,2-dimethylpropionic acid (C5), caproic acid (C6;hexanoic acid), 2,2-dimethylbutyric acid(C6), 2-ethylbutyric acid (C6), and 2-methylvaleric acid (C6; 2-methylpentanoic acid) were purchased from Sigma-Aldrich (St. Louis, MO, USA). Isotope-labeled internal standards, including acetic acid-d_3_ (C2-2,2,2-d_3_), propionic acid-d_6_ (C3-d_6_), butyric acid-d_7_ (C4-d_7_), valeric acid-d_4_ (C5-2,2,3,3-d_4_), and caproic acid-d_5_ (C6-5,5,6,6,6-d_5_), were purchased from Sigma-Aldrich (St. Louis, MO). All of the stock solutions were prepared in water and stored at −20 °C. 4-Acetoamido-7-mercapto-2,1,3-benzoxadiazole (AABD-SH) was purchased from Tokyo Chemical Industry Co., Ltd (Tokyo, Japan). Triphenylphosphine (TPP), 2,2’-dipyridyl disulfide (DPDS), and other reagents, including mobile phase solvents, were from Sigma-Aldrich or J. T. Bakers (Center Valley, PA, USA).

### 2.2. Sample Preparation

380 μL water and 20 μL internal standard solution (10 μM each of five internal standards in water) were added to 20–25 mg mouse feces, and the feces sample was homogenized while using a tissueLyzer (Qiagen Inc., Valentia, CA, USA) at 30 Hz for 1.5 min. The sample was centrifuged at 18,900× *g* for 10 min. at 4 °C, and the supernatant was collected. Afterwards, the solution was transferred to membrane filter (Nanosep 3K centrifugal device, Omega membrane, Pall Corporation, New York, USA) and it was centrifuged at 19,000× *g* for 20 min. to remove any floating particulates in the supernatant.

For plasma, 380 μL water and 20 μL internal standard solution were added to 200 μL mouse plasma, and mixed well. For exhaled breath condensate, 20 μL internal standard solution was added to 1 mL human exhaled breath condensate (EBC), and mixed well. The mixtures were centrifuged at 19,500× *g* for 10 min. at 4 °C, and the supernatants were then collected. 

For derivatization of SCFAs, 20 μL each of 20 mM AABD-SH, 20 mM TPP, and 20 mM DPDS in dichloromethane were added to the supernatant of a biological sample in a glass tube, and derivatization was performed at room temperature for 5 min while vortexing. The reaction solution was dried under vacuum, and then reconstituted with 20 μL methanol prior to LC-MS/MS analysis. The calibration curves were generated with standard solutions (100 nM to 1 mM).

### 2.3. Biological Samples

Mice were housed at 21–23 °C with 12 h light/12 h dark cycles at the SPF Animal Facility at the University of Ulsan, with free access to water and rodent chow. All of the animal care and procedures were conducted according to the protocols and guidelines that the University of Ulsan Animal Care and Use Committee approved. Mice feces were collected from 23-week-old male C57BL/6 mice fed a high-fat chow (60% kcal% fat, Research diets, Inc., New Brunswick, NJ, USA) or low-fat chow (10% kcal% fat, Research diets, Inc.) for 12 weeks. Mice feces were frozen in liquid nitrogen and stored at −80 °C until processing. Mice plasma were prepared after anesthesia of male C57BL/6 mice at eight weeks of age. Human EBCs were obtained while using RtubeTM (Respiraotry Research Inc., Austin, TX), following manufacturer’s instruction. The study protocol was approved by the Institutional Review Board (IRB) of Asan Medical Center (2018-0789).

### 2.4. Liquid Chromatography-Tandem Mass Spectrometry (LC-MS/MS)

An LC-MS/MS system that was equipped with a 1290 HPLC instrument (Agilent Technologies, Glostrup, Denmark), a QTRAP 5500 (ABSciex, Framingham, MA, USA), and a reversed-phase column (Pursuit 5 C18 150 × 2.0 mm; Agilent Technologies, Santa Clara, USA) was employed. MS was conducted in positive ion mode with a turbo ion-spray voltage of 5500 V, while using 20 psi curtain gas, 50 psi nebulizer gas, and 50 psi drying gas at a temperature of 400 °C. The sample injection volume was 3 µL. LC separation was performed while using mobile phase A (0.1 % formic acid in water) and mobile phase B (0.1% formic acid in acetonitrile), at a flow rate of 500 µL/min and a temperature of 40 °C. The separation gradient was as follows: 30% B at 0 min., 30 to 50% B in 30 min., 50 to 30% B in 0.1 min., and 30% B in 4.9 min. A collision energy of 15 V was used for multiple reaction monitoring (MRM), and LC-MS/MS data were analyzed by Analyst 1.5.2 software (AB Sciex). Peak area of each isotope-labeled internal standard was used to normalize that of straight or branched SCFA having the same number of carbons.

### 2.5. Method Validation

LOQ (limit of quantitation) was obtained from a calibration curve and calculated while using the formula 10 × S_a_/b, where S_a_ is the standard deviation of the Y-intercept of linear regression line, and b is the slope of the calibration curve. [15] Intra- and inter-day reproducibility for standard solutions and biological samples were expressed as relative standard deviation (RSD, %) of three measurements. For intra-day reproducibility, a sample was analyzed three times on the same day. For inter-day reproducibility, a sample was repeatedly analyzed on three consecutive days, while the solution was stored at 4 °C. The quantitation accuracy was assessed by comparing the amount of each SCFA in the spiked and non-spiked supernatants. The known amount of SCFAs was spiked into half of the supernatant of a biological sample after prepared, as described above, and the remaining half of the supernatant was used as the non-spiked control. Additionally, the amounts of spiked SCFAs in the supernatant were expressed as molar concentrations. Quantitation accuracy (%) was calculated while using the equation of (the measured amount of SCFA in spiked supernatant—the measured amount of SCFA in non-spiked control) ÷ the known amount of spiked SCFA in spiked supernatant × 100. The stability of derivatizatives was determined by the repeated injection of the sample solution on day 1, 5, and 7, while they were kept at 4 °C. On each day, three measurements were performed.

## 3. Results and Discussion

### 3.1. Liquid Chromatography-Tandem Mass Spectrometry (LC-MS/MS) of Derivatized Short Chain Fatty Acids

SCFAs were derivatized with AABD-SH. This compound has been used as a fluorescent derivatization reagent for carboxylic acid moieties, such as fatty acids, bile acids, and prostaglandins [16]. Biological samples contain many kinds of carboxylic acids, hence MS is suitable for the specific detection of biomolecules derivatized with AABD-SH. Derivatized SCFAs with AABD-SH resulted in reasonable chromatographic separation and good electrospray ionization (ESI). The specific detection of each SCFA was possible from the unique fragmentation pattern during collision-induced dissociation (CID). Derivatized SCFAs could be separated while using a reversed-phase LC column, and different retention times among 12 SCFAs provide another feature for specific analysis. 

**Figure 1:**
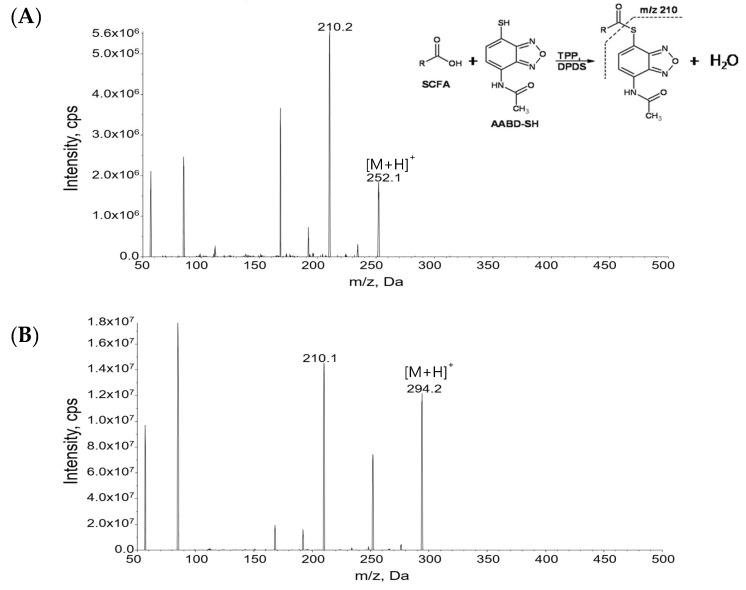
Tandem mass spectrometry (MS/MS) spectra of (**A**) derivatized acetic acid (C2) and (**B**) derivatized valeric acid (C5). Collision energies were 15 V.

**Figure 2:**
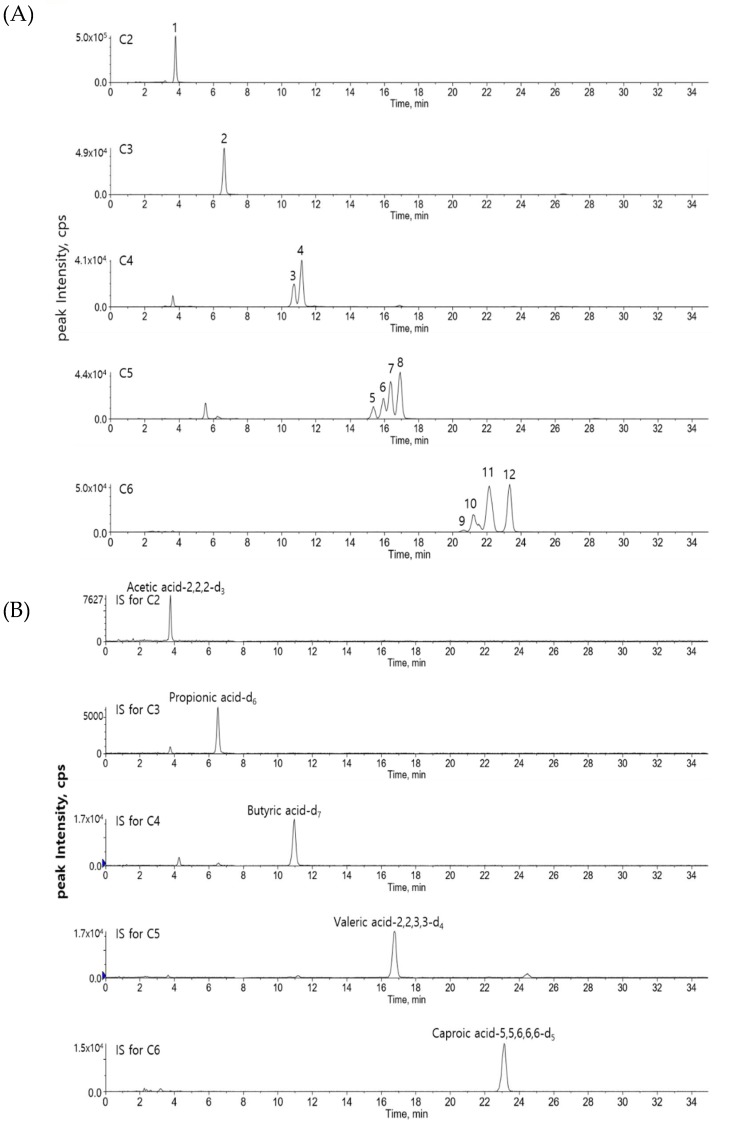
Extracted ion chromatograms of (**A**) SCFAs and (**B**) internal standards in a standard solution (10 μM). The number on top of each peak represents each SCFA (1: acetic acid; 2: propionic acid; 3: isobutyric acid; 4: butyric acid; 5: 2,2-dimethylpropionic acid; 6: 2-methylbutyric acid; 7: isovaleric acid; 8: valeric acid; 9: 2,2-dimethylbutyric acid; 10: 2-ethylbutyric acid; 11: 2-methylvaleric acid; 12: caproic acid). IS represents internal standard.

Figure 1 shows MS/MS spectra of derivatized acetic acid (C2) and valeric acid (C5). The most abundant product ion in MS/MS was *m/z* 210.2, corresponding to the derivatization reagent, hence this peak was used for quantitation. For isotope-labeled internal standards of acetic acid, propionic acid, butyric acid, and valeric acid, the product ion was *m/z* 211.2, due to the presence of deuterium at C-2 position of carboxylic acid (Figure S1), where the deuterium was transferred to thiol group of a derivatization reagent when it was cleavaged during CID process. Internal standard of caproic acid does not have any deuterium at C-2 position of carboxylic acid, and the product ion during CID was *m/z* 209.8. Table 1 summarizes the instrumental parameters, MS/MS transitions, and analytical parameters of this method. Figure 2 shows the extracted ion chromatogram (EIC) of each SCFA and its corresponding internal standard (EICs for 12 SCFAs in various biological samples can be found in Figure S2). The derivatization of SCFAs with AABD-SH increased both the size and hydrophobicity of the molecules, and it resulted in longer retention times on reversed-phase LC. SCFAs with 4–6 carbons consist of several structural isomers. This strategy afforded good separation of structural isomers. Shorter SCFAs eluted earlier than longer SCFAs, and straight chain SCFAs eluted later than the branched chain SCFAs, as expected.

### 3.2. Optimization of the Derivatization Reaction and Method Validation 

**Figure 3:**
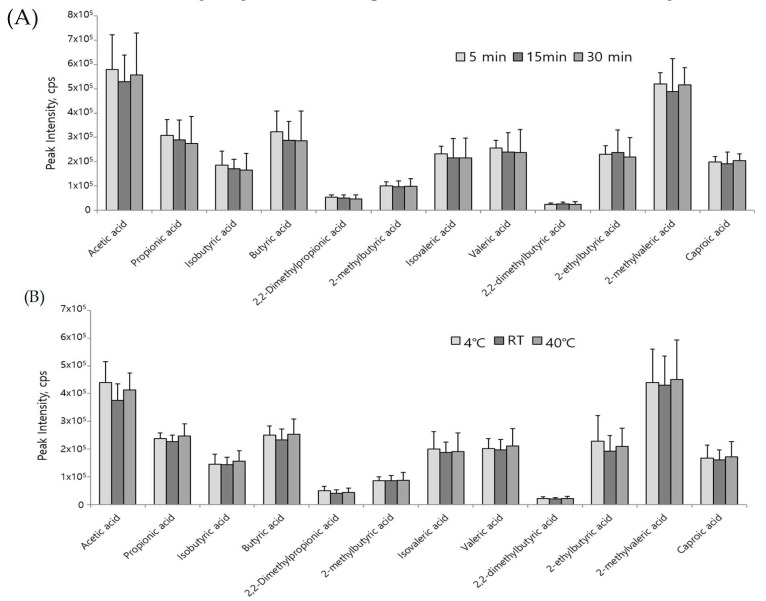
Optimization of derivatization reaction in terms of (**A**) reaction time and (**B**) reaction temperature. Optimized reaction conditions were evaluated using three independent determinations of SCFAs in mouse feces on each condition. Error bar represents the standard deviation of three measurements. RT represents room temperature.

**Figure 4:**
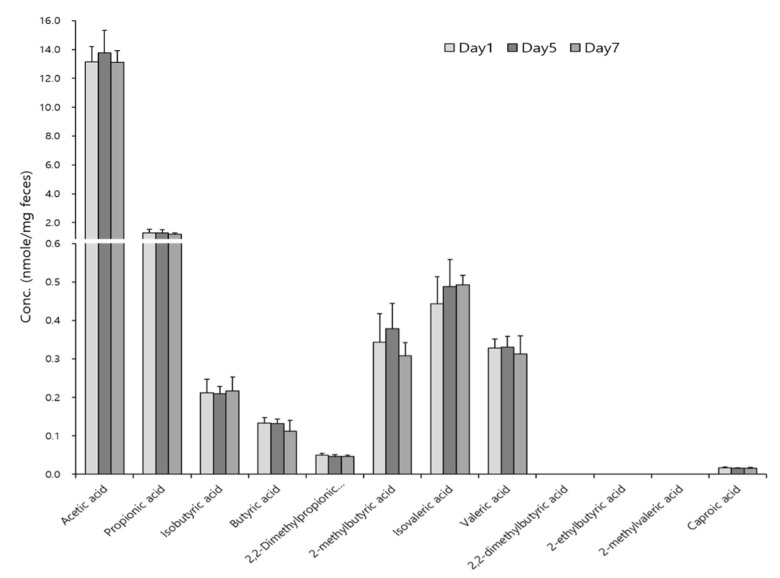
Stability of derivatized SCFAs in mouse feces. Sample solutions containing derivatized SCFAs were stored at 4 °C and measured on day 1, 5, and 7. Three measurements were performed on each day. Error bar represents the standard deviation of three measurements.

The reaction duration was optimized first at room temperature and it was not a critical factor for derivatization (Figure 3A). Next, we investigated the effect of reaction temperature on derivatization, and the differences of peak abundances at different temperatures (4 °C, room temperature, and 40 °C) were negligible (Figure 3B). Thus, chemical derivatization was performed for 5 min. at room temperature in subsequent experiments. The main advantage of the method is that the derivatization reaction duration is short, and the conditions are mild (room temperature) and barely affect the experimental results. The derivatized reaction products were stable for a week (Figure 4). The calibration curve for each SCFA can be found in Figure S3, and analytical parameters, such as LOQ, accuracy, and reproducibility, are shown in Table 1. The reproducibility of this method was also evaluated with biological samples (Table 2). RSD was less than 10 % for intra- and inter-day measurements with few exceptions. 

Quantitation accuracy was determined with the amount of recovered SCFA after spiking known amount of standard solutions into the supernatants of biological samples (Table S1). The quantitation accuracies for straight chain SCFAs ranged from 85 to 114%. However, the quantitation accuracies for branched chain SCFAs ranged from 51 to 130 %. The internal standards used for this study were all isotope-labeled straight chain SCFAs, thus use of isotope-labeled branched chain SCFAs may improve the quantitation accuracies for branched chain SCFAs. Other research groups also reported similar observations [17,18]. To best of our knowledge, this is the first measurement of SCFAs in human exhaled breath condensates (EBCs), although only several SCFAs were observed. Recently, the role of the microbiome in pulmonary diseases has drawn attention, and SCFAs have been determined in diverse airway samples, such as sputum and bronchoalveolar lavage [19,20,21]. Thus, we suspected that SCFAs would be present in human EBCs. The determination of SCFAs in EBCs could be useful for investigating the relationships between the microbiome and lung diseases.

This simple and robust derivatization reaction facilitates LC-MS/MS analysis of SCFAs. However, LC separation among structural isomers of C5 SCFAs would be improved, as shown in Figure S4, where the complete baseline separation for 12 SCFAs was achieved with less steep gradient and lower column temperature in longer LC run time (65 min.). Instead, we chose shorter LC run time (35 min.), as shown in Figure 2, although baseline separation between peak 6 and peak 7 was not achieved. Resolution (*R*_s_) between two peaks is defined as the difference in the retention times between two peaks, divided by the combined widths of the two peaks (2 × (t_R2_ − t_R1_) / (w_1_ + w_2_)) and R_s_ between peak 6 and peak 7 in Figure 2 was 1. A study reported that the integration error was less than about 2% for *R*_s_ = 1 when a smaller peak is about 60 % of a larger peak, and drop method and peak area were used for quantitation [22,23]. In our study, the peak height of peak 6 was approximately 60 % of that of peak 7, and we also used drop method and peak area for quantitation. Thus, the quantitation error for peak 6 and peak 7 may not be serious, although it should depend on the type of your research. The integration error could be decreased further if peak height was used instead of peak area for quantitation [22]. If more accurate measurement was necessary for all structural isomers of C5 SCFAs, experimental condition used in Figure S4 should be used. Analysis of large number of samples is often required in biological studies. Only small number of samples could be analyzed in a batch when 65 min. LC run time is used, considering analysis of calibration solutions and QC samples in every batch. For high throughput analysis, experimental conditions using 35 min. LC run time was chosen in our study. Alternatively, C8 reversed-phase column may perform similarly as C18 reversed-phase column in a shorter run time, although a significant effort needs to optimize chromatographic condition [24,25]. Various kinds of metabolites in biological samples often contain carboxylic acids. Thus, the precursor ion scan of 210 for derivatized metabolites with AABD-SH would be useful for profiling metabolites with carboxylic acid moiety in metabolomics studies.

### 3.3. Profiling of SCFAs in Biological Samples 

The developed method was applied to profile SCFAs in mice feces of high-fat or low-fat diet shown in Table 3. Lower acetic acid and propionic acid were observed in the mice feces of high-fat diet. However, butyric acid and valeric acid were increased in high-fat diet mice. One research group showed that fecal acetic acid, propionic acid, and butyric acid were decreased in high-fat diet when compared to low fat diet [26]. Another research group reported that dietary fat resulted in decreased acetic acid, propionic acid, and butyric acid in cecum content of pigs [27]. Fecal SCFAs of high-fat diet mice were also compared with those of normal diet mice. High-fat diet induced significant weight gain and reduced fecal SCFAs [28,29]. These studies generally reported that high-fat diet induced decreased fecal SCFAs. In our study, more than half of the fecal SCFAs were reduced in high fat diet as compared to low fat diet. Variations in fecal SCFAs may be attributed to the specific experimental conditions, such as the amount of fat intake, feeding periods, and ages of mice. 

## 4. Conclusions

A simple profiling method of SCFAs using LC-MS/MS was developed. The derivatization reaction was finished for 5 min. at room temperature. Good accuracy and reproducibility were obtained at various biological matrices. This method can facilitate the profiling of SCFAs in various biological samples, in which the SCFA content is highly variable due to diet or microbiome.

## Figures and Tables

**Table 1:** Instrumental parameters, MS/MS transitions used in multiple reaction monitoring (MRM) mode, and analytical parameters, including LOQ (limit of quantitation), accuracy, and reproducibility were shown (*n* = 3). CE (collision energy) and CXP (collsion cell exit potential) for all short chain fatty acids (SCFAs) and internal standards were 15 V and 20 V, respectively, except CXP (10 V) for acetic acid and propionic acid. DP represents declustering potential, and EP represents entrance potential. R^2^ is correlation coefficient of a calibration curve. IS represents internal standard.

Compound	DP	EP	MRM Transitions		IS	Calibration Curve		LOQ, μM (ng/mL)	Intraday (10 μM)	Interday (10 μM)
	(V)	(V)	Q1	Q3		Calibration Range (μM)	R^2^		RSD, % (Accuracy, %)	RSD, % (Accuracy, %)
Acetic acid	70	8	252	210	A	1–1000	1	1.03 (61.8)	6.66 (98.5)	14.9 (103)
Propionic acid	30	10	266	210	B	0.1–1000	0.999	0.293 (21.7)	0.909 (110)	15.1 (97.4)
Isobutyric acid	70	6	280	210	C	0.1–1000	0.999	0.450 (39.6)	1.05 (110)	7.97 (93.2)
Butyric acid	70	6	280	210	C	0.1–1000	1	0.158 (13.9)	1.72 (116)	13.4 (97.0)
2,2-dimethylpropionic acid	50	10	294	210	D	0.1–100	0.989	0.470 (47.9)	1.75 (110)	11.3 (86.1)
2-Methylbutyric acid	50	10	294	210	D	0.1–500	0.998	0.773 (78.8)	1.50 (102)	8.87 (86.9)
Isovaleric acid	50	10	294	210	D	0.1–100	0.999	0.624 (63.6)	2.11 (103)	9.09 (91.4)
Valeric acid	50	10	294	210	D	0.1–1000	0.997	0.451 (46.0)	0.475 (101)	6.00 (87.0)
2,2-Dimethylbutyric acid	70	10	308	210	E	0.1–100	0.997	0.430 (49.9)	7.67 (116)	11.6 (85.7)
2-Ethylbutyric acid,	70	10	308	210	E	0.1–100	0.999	0.546 (63.3)	4.83 (108)	12.2 (96.0)
2-Methylvaleric acid	70	10	308	210	E	0.1–500	0.983	0.773 (89.7)	3.38 (112)	4.49 (105)
Caproic acid	70	10	308	210	E	0.1–1000	0.994	0.609 (70.6)	1.66 (89.8)	4.74 (89.3)
Acetic acid-2,2,2-d_3_ (A)	70	6	255	211	-	-	-	-	-	-
Propionic acid-d_6_ (B)	30	8	272	211	-	-	-	-	-	-
Butyric acid- d_7_ (C)	70	8	287	211	-	-	-	-	-	-
Valeric acid-2,2,3,3-d_4_ (D)	50	8	298	211	-	-	-	-	-	-
Caproic-5,5,6,6,6-d_5_ (E)	70	8	313	210	-	-	-	-	-	-

**Table 2:** Average amounts of SCFAs in biological samples, and their intra- and inter-day reproducibilities. ND represents “not detected”. (*n* = 3 for intra- or inter-day reproducibility)**.**

	Mouse Feces (nmol/mg)			Mouse plasma (μM)			Human EBC (μM)		
	Average	Intraday	Interday	Average	Intraday	Interday	Average	Intraday	Interday
		RSD (%)	RSD (%)		RSD (%)	RSD (%)		RSD (%)	RSD (%)
Acetic acid	19.5	3.25	8.04	77.4	1.61	8.03	66.4	1.27	6.02
Propionic acid	3.54	0.866	2.88	8.08	3.1	2.27	13.4	1.17	2.41
Isobutyric acid	0.802	4.87	9.23	31.1	7.37	9.74	2.43	5.2	6.15
Butyric acid	0.0934	8.13	11.2	20.7	5.46	8.98	2.79	6.01	2.28
2,2-Dimethylpropionic acid	ND	-	-	35.9	5.17	9.73	ND	-	-
2-Methylbutyric acid	0.645	1.12	10	14.3	6.54	12.5	ND	-	-
Isovaleric acid	0.379	1.29	8.84	9.92	6.95	18	ND	-	-
Valeric acid	0.224	1.64	4.53	6.16	10.4	11.5	ND	-	-
2,2-Dimethylbutyric acid	ND	-	-	ND	-	-	ND	-	-
2-Ethylbutyric acid	ND	-	-	0.651	7.36	11.1	ND	-	-
2-Methylvaleric acid	ND	-	-	2.09	6.07	2.01	ND	-	-
Caproic acid	0.0125	8.74	9.61	0.711	6.66	8.95	ND	-	-

**Table 3:** Quantitation of SCFAs in mice feces fed with high-fat (HFD) or low-fat (LFD) diet. Averages and STDs (standard deviations) of SCFAs were calculated by using three feces samples taken from three mice. ** indicates if *p*-value < 0.01 and * if *p* < 0.05 (*t* test). ND represents “not detected”.

	HFD Mouse Feces, (nmol/mg)		LFD Mouse Feces, (nmol/mg)		*t* Test
	Average	STD	Average	STD	
Acetic acid	16.4	0.659	19.5	1.57	*
Propionic acid	2.99	0.118	3.54	0.102	**
Isobutyric acid	0.416	0.0655	0.802	0.074	**
Butyric acid	0.821	0.0352	0.0934	0.0105	**
2,2-dimethylpropionic acid	ND	-	ND	-	
2-Methylbutyric acid	0.534	0.0503	0.645	0.0646	
Isovaleric acid	0.675	0.0046	0.379	0.0335	**
Valeric acid	1.389	0.0698	0.223	0.0101	**
2,2-Dimethylbutyric acid	ND	-	ND	-	
2-Ethylbutyric acid	ND	-	ND	-	
2-Methylvaleric acid	ND	-	ND	-	
Caproic acid	0.00478	0.000357	0.0125	0.0012	**

## Supplementary Materials

The following are available online at https://www.mdpi.com/2218-1989/9/9/173/s1, Table S1: Quantitation accuracies of spiked SCFAs in mouse feces, mouse plasma and human EBC. Figure S1: Tandem mass spectrometry (MS/MS) spectra of (A) derivatized acetic acid-2,2,2-d_3_ (internal standard for C2), (B) derivatized valeric acid-2,2,3,3-d_4_ (internal standard for C5), (C) derivatized Caproic acid-5,5,6,6,6-d_5_ (internal standard for C6), Figure S2: Extracted ion chromatograms of SCFAs in (A) a standard solution (1μM) and (B) mouse feces (C) mouse plasma, and (D) human EBC, Figure S3: Calibration curves for 12 SCFAs, Figure S4: Extracted ion chromatograms of 12 SCFAs in a standard solution (10 μM, 65 min. LC run time).
