# Supplementary material for: A Facile Profiling Method of Short Chain Fatty Acids Using Liquid Chromatography-Mass Spectrometry
Source: Metabolites. 2019 Aug 28;9(9):173. doi: 10.3390/metabo9090173 (PMC6780976; doi:10.3390/metabo9090173)
Supplement: Supplementary file 1 [file metabolites-09-00173-s001.pdf]

Supplementary information

A Facile Profiling Method of Short Chain Fatty Acids using Liquid Chromatography-Mass Spectrometry

Ha Eun Song[1], Hyo Yeong Lee[1], Su Jung Kim[1], Sung Hoon Back[2], and Hyun Ju Yoo[1*]

[1] Department of Convergence Medicine, Asan Institute for Life Sciences, Asan Medical Center, University of Ulsan College of Medicine, Seoul 05505, Republic of Korea; hes1290@snu.ac.kr (H.E.S.); lhy_1279@naver.com (H.Y.L.); su-jungea@hanmail.net (S.J.K.); yoohyunju@amc.seoul.kr (H.J.Y.)

[2] School of Biological Sciences, University of Ulsan, Ulsan 44610, Republic of Korea; shback@ulsan.ac.kr (S.H.B.)

* Correspondence: yoohyunju@amc.seoul.kr; Tel: 82-02-3010-4029

Supplemenatry Table

Matrix	Analyte	Amount spiked (μM)	Amount recovered (μM)	Quantitation accuracy (%)	
				Average	RSD
Mouse feces	Acetic acid	500	-	-	-
	Propionic acid	500	580	116	14
	Isobutyric acid	500	508	102	6
	Butyric acid	500	553	111	1
	2,2-Dimethylpropionic acid	500	255	51	12
	2-Methylbutyric acid	500	373	75	12
	Isovaleric acid	500	256	51	12
	Valeric acid	500	424	85	6
	2,2-Dimethylbutyric acid	500	259	52	16
	2-Ethylbutyric acid	500	252	50	8
	2-Methylvaleric acid	500	265	53	8
	Caproic acid	500	441	88	2
Mouse plasma	Acetic acid	100	92	92	1
		500	507	101	6
	Propionic acid	100	87	87	7
		500	554	111	16
	Isobutyric acid	100	120	120	8
		500	650	130	4
	Butyric acid	100	98	98	5
		500	569	114	2
	2,2-Diethylpropionic acid	100	87	87	9
		500	391	78	9
	2-Methylbutyric acid	100	118	118	11
		500	510	102	3
	Isovaleric acid	100	78	78	2
		500	343	69	11
	Valeric acid	100	88	88	4
		500	424	85	2
	2,2-Dimethylbutyric acid	100	78	78	6
		500	593	119	5
	2-Ethylbutyric acid	100	61	61	2
		500	254	51	14
	2-Methylvaleric acid	100	80	80	4
		500	308	62	9
	Caproic acid	100	102	102	6
		500	427	85	9
Human EBC	Acetic acid	100	81	81	-
		500	533	107	-
	Propionic acid	100	88	88	-
		500	540	108	-
	Isobutyric acid	100	94	94	-
		500	458	92	-
	Butyric acid	100	82	82	-
		500	469	94	-
	2,2-Diethylpropionic acid	100	105	105	-
		500	472	94	-
	2-Methylbutyric acid	100	69	69	-
		500	307	61	-
	Isovaleric acid	100	87	87	-
		500	408	82	-
	Valeric acid	100	113	113	-
		500	572	114	-
	2,2-Dimethylbutyric acid	100	75	75	-
		500	509	102	-
	2-Ethylbutyric acid	100	52	52	-
		500	396	79	-
	2-Methylvaleric acid	100	104	104	-
		500	447	89	-
	Caproic acid	100	97	97	-
		500	572	114	-

Table S1. Quantitation accuracies of spiked SCFAs in mouse feces, mouse plasma and human EBC. Quantitation accuracy (%) of each SCFA was calculated using the equation of (the measured amount of SCFA in spiked supernatant – the measured amount of SCFA in non-spiked control) ÷ the known amount of spiked SCFA in spiked supernatant × 100. Three independent measurements were used, except human EBC where only one measurement was performed. Mouse feces already contain significant amount of acetic acid, thus acetic acid was not included in the spiking solution because it will exceed dynamic range. Amount spiked (μM) means "the known amount of spiked SCFA in spiked supernatant", and amount recovered (μM) means "the measured amount of SCFA in spiked supernatant – the measured amount of SCFA in non-spiked control".

Supplementary Figures

(A)

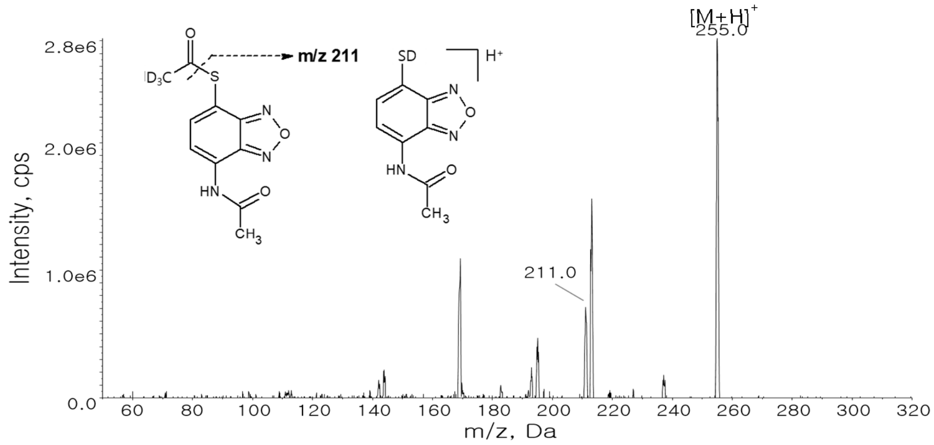

(B)

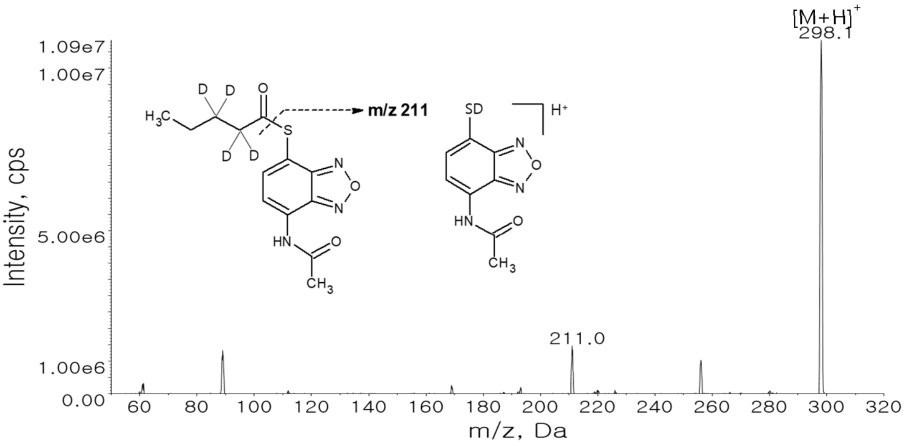

(C)

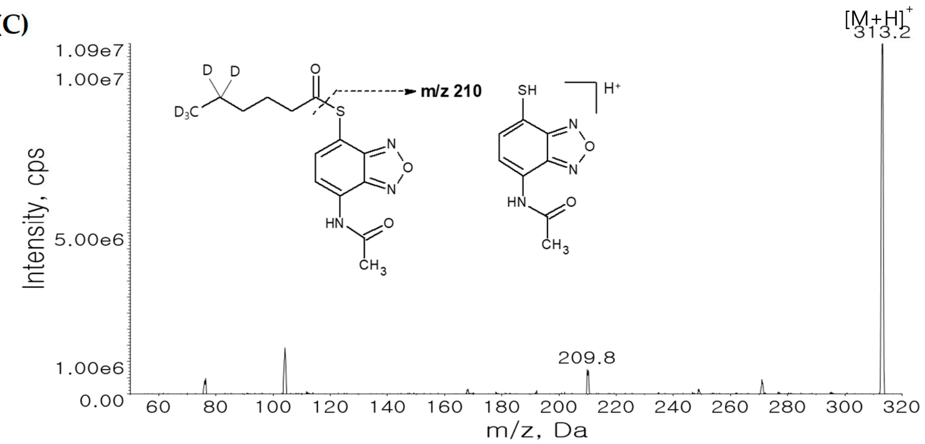

Figure S1. Tandem mass spectrometry (MS/MS) spectra of (A) derivatized acetic acid-2,2,2-d3 (internal standard for C2), (B) derivatized valeric acid-2,2,3,3-d4 (internal standard for C5), (C) derivatized Caproic acid-5,5,6,6,6-d5 (internal standard for C6). Collision energies were 15 V.

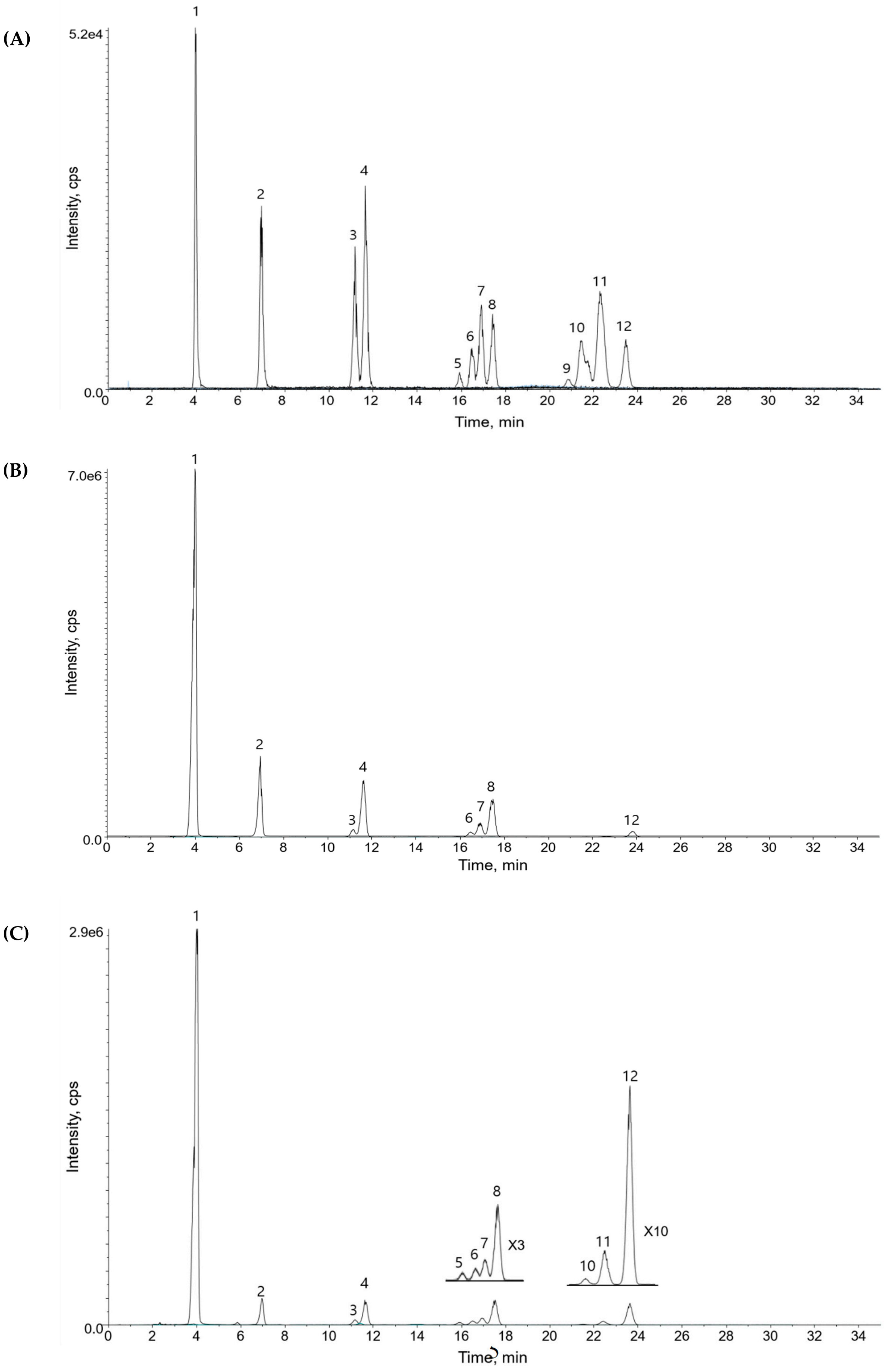
(A)
5.2e4
Intensity, cps
0.0
0 2 4 6 8 10 12 14 16 18 20 22 24 26 28 30 32 34
Time, min
1 2 3 4 5 6 7 8 9 10 11 12
(B)
7.0e6
Intensity, cps
0.0
0 2 4 6 8 10 12 14 16 18 20 22 24 26 28 30 32 34
Time, min
1 2 3 4 6 7 8 12
(C)
2.9e6
Intensity, cps
0.0
0 2 4 6 8 10 12 14 16 18 20 22 24 26 28 30 32 34
Time, min
1 2 3 4 5 6 7 8 X3 10 11 12 X10

(D)

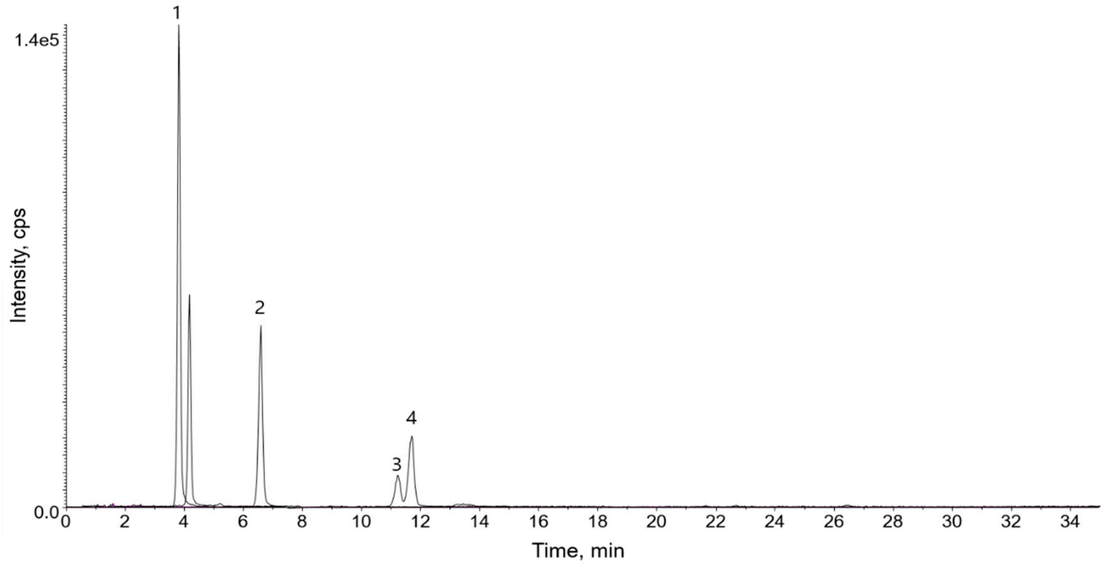

Figure S2. Extracted ion chromatograms of SCFAs in (A) a standard solution (1μM) and (B) mouse feces (C) mouse plasma, and (D) human EBC. The number on top of each peak represents each SCFA. (1: acetic acid; 2: propionic acid; 3: isobutyric acid; 4: butyric acid; 5: 2,2-dimethylpropionic acid; 6: 2-methylbutyric acid; 7: isovaleric acid; 8: valeric acid; 9: 2,2-dimethylbutyric acid; 10: 2-ethylbutyric acid; 11: 2-methylvaleric acid; 12: caproic acid)

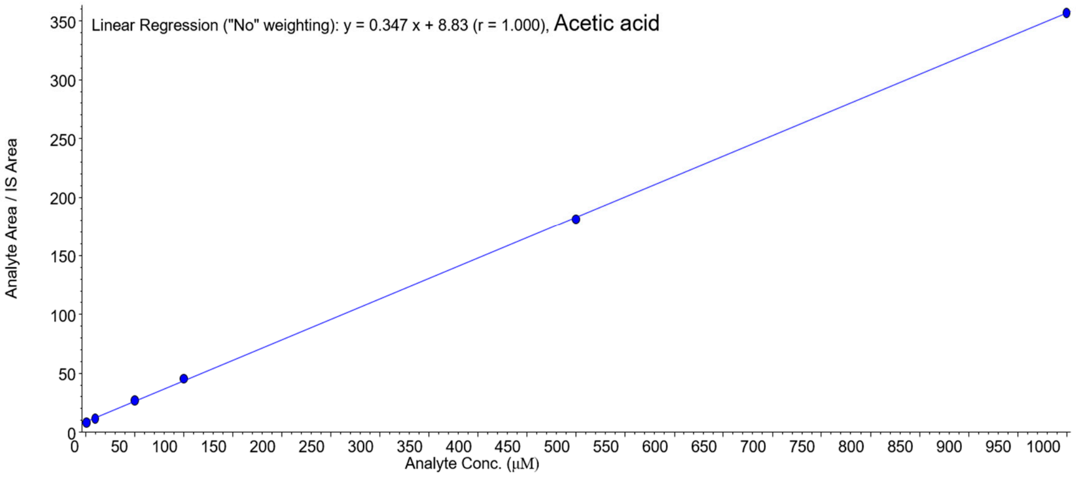
Linear Regression ("No" weighting): y = 0.347 x + 8.83 (r = 1.000), Acetic acid
Analyte Area / IS Area
Analyte Conc. (μM)

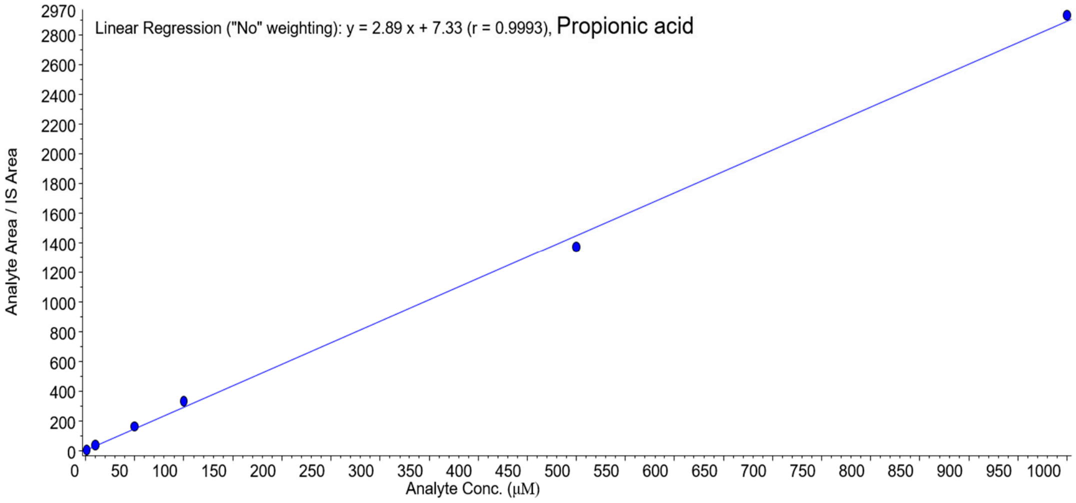
Linear Regression ("No" weighting): y = 2.89 x + 7.33 (r = 0.9993), Propionic acid
Analyte Area / IS Area
Analyte Conc. (μM)

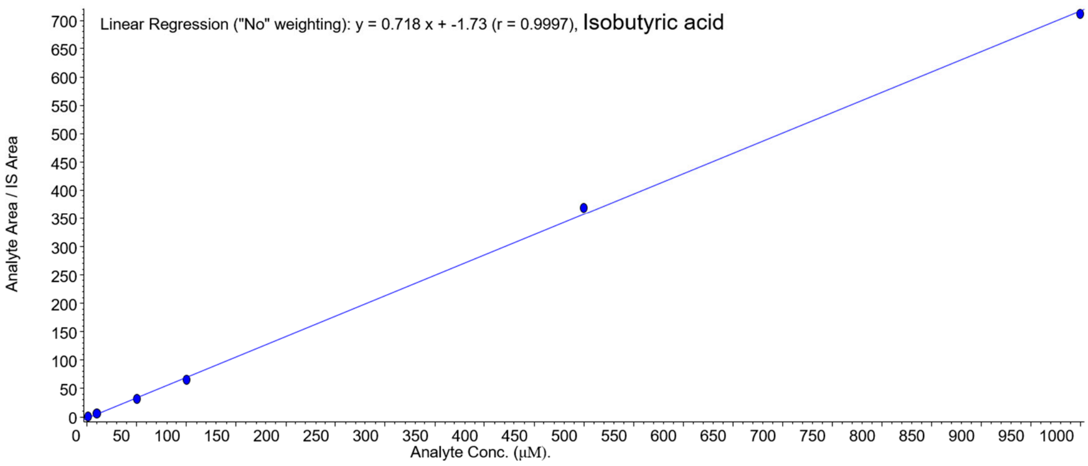
Linear Regression ("No" weighting): y = 0.718 x + -1.73 (r = 0.9997), Isobutyric acid
Analyte Area / IS Area
Analyte Conc. (μM).

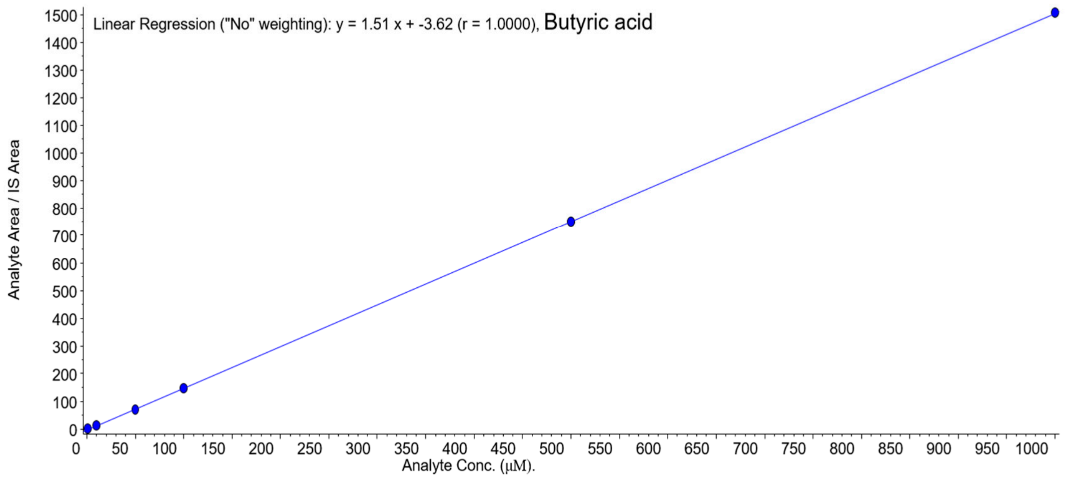
Linear Regression ("No" weighting): y = 1.51 x + -3.62 (r = 1.0000), Butyric acid
Analyte Area / IS Area
Analyte Conc. (μM).
0
100
200
300
400
500
600
700
800
900
1000
1100
1200
1300
1400
1500
0
50
100
150
200
250
300
350
400
450
500
550
600
650
700
750
800
850
900
950
1000

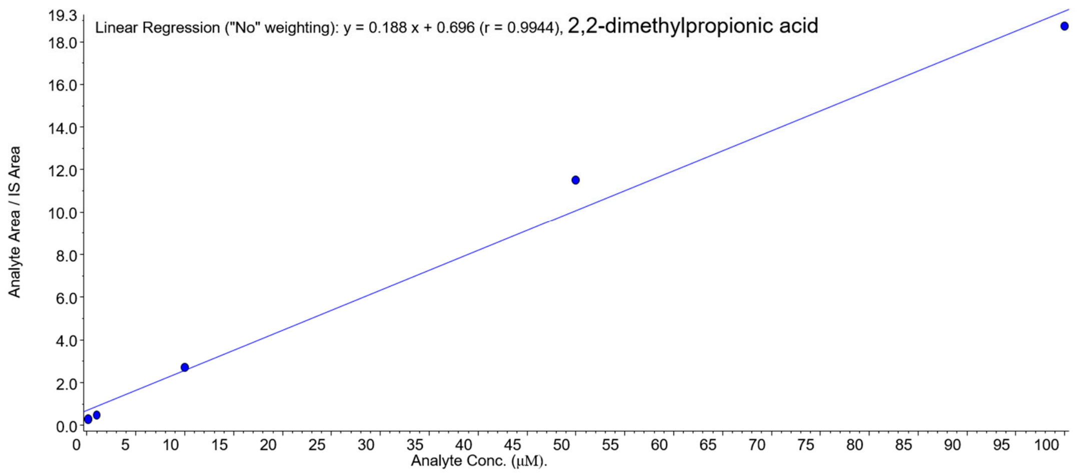
Linear Regression ("No" weighting): y = 0.188 x + 0.696 (r = 0.9944), 2,2-dimethylpropionic acid
Analyte Area / IS Area
Analyte Conc. (μM).
0.0
2.0
4.0
6.0
8.0
10.0
12.0
14.0
16.0
18.0
19.3
0
5
10
15
20
25
30
35
40
45
50
55
60
65
70
75
80
85
90
95
100

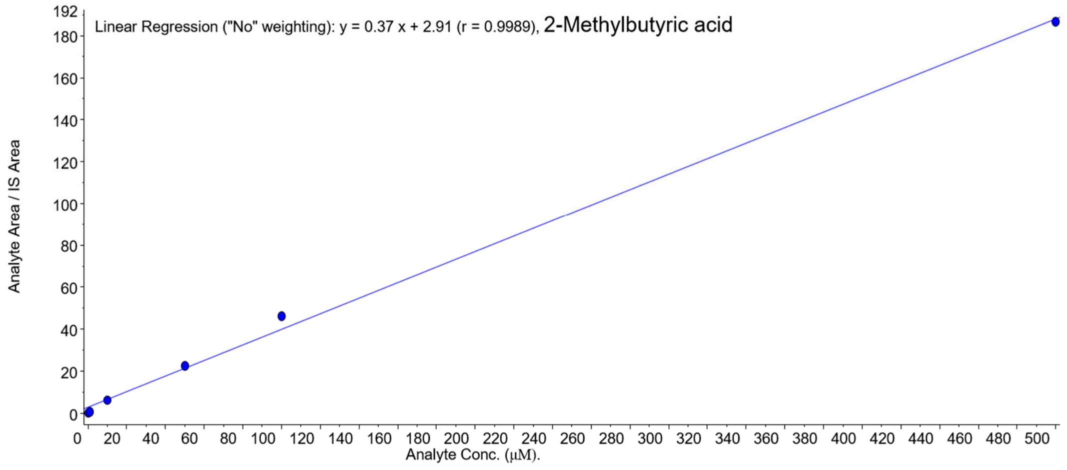
Linear Regression ("No" weighting): y = 0.37 x + 2.91 (r = 0.9989), 2-Methylbutyric acid
Analyte Area / IS Area
Analyte Conc. (μM).
0
20
40
60
80
100
120
140
160
180
192
0
20
40
60
80
100
120
140
160
180
200
220
240
260
280
300
320
340
360
380
400
420
440
460
480
500

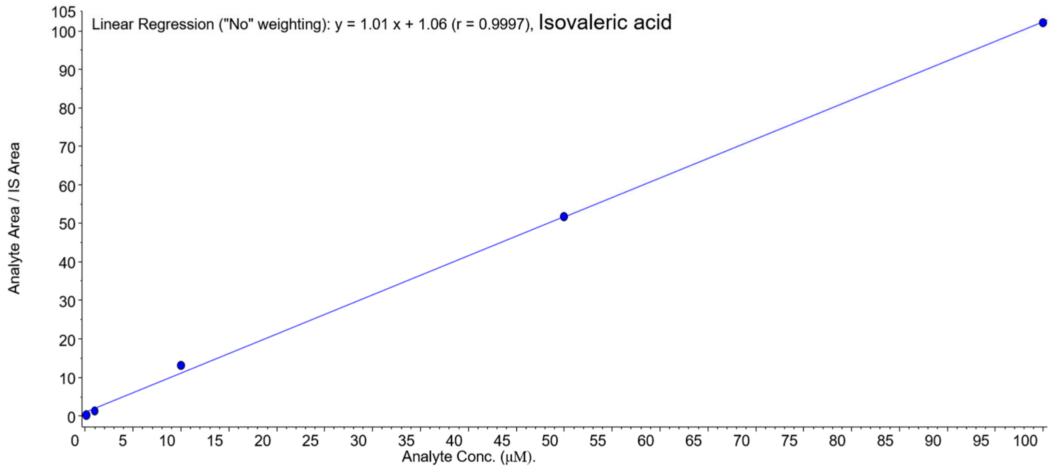
Linear Regression ("No" weighting): y = 1.01 x + 1.06 (r = 0.9997), Isovaleric acid
Analyte Area / IS Area
Analyte Conc. (μM).

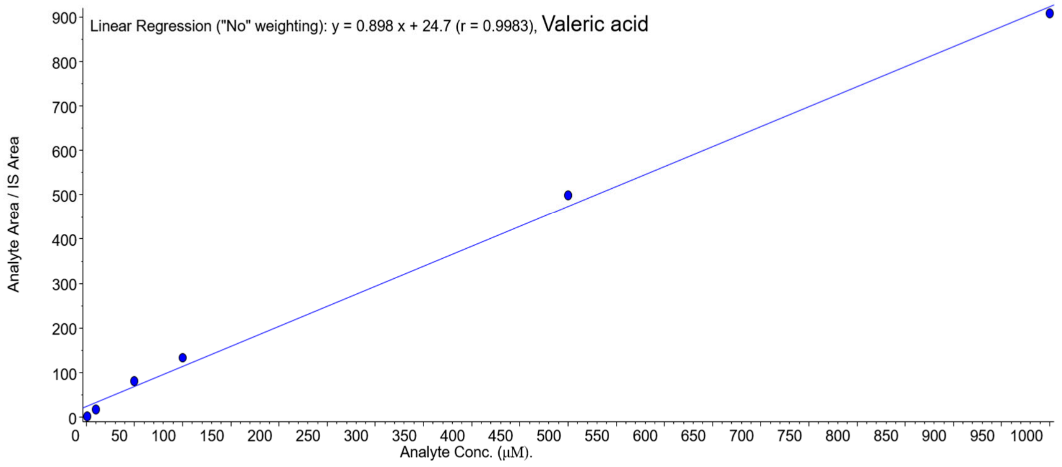
Linear Regression ("No" weighting): y = 0.898 x + 24.7 (r = 0.9983), Valeric acid
Analyte Area / IS Area
Analyte Conc. (μM).

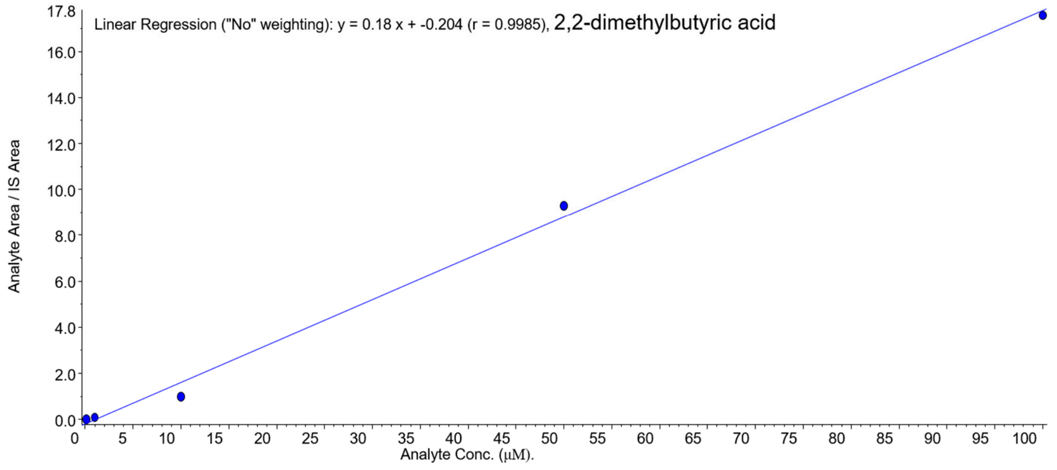
Linear Regression ("No" weighting): y = 0.18 x + -0.204 (r = 0.9985), 2,2-dimethylbutyric acid
Analyte Area / IS Area
Analyte Conc. (μM).

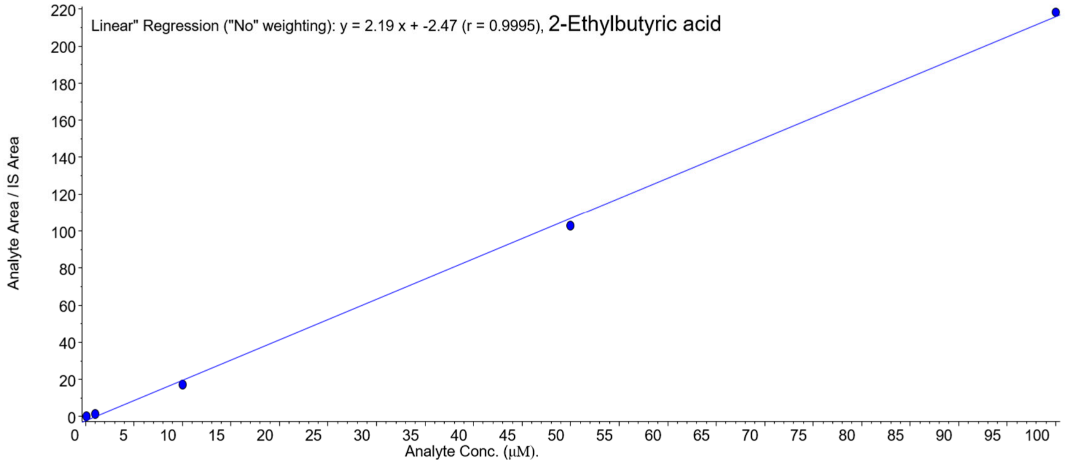
Linear" Regression ("No" weighting): y = 2.19 x + -2.47 (r = 0.9995), 2-Ethylbutyric acid
Analyte Area / IS Area
Analyte Conc. (µM).

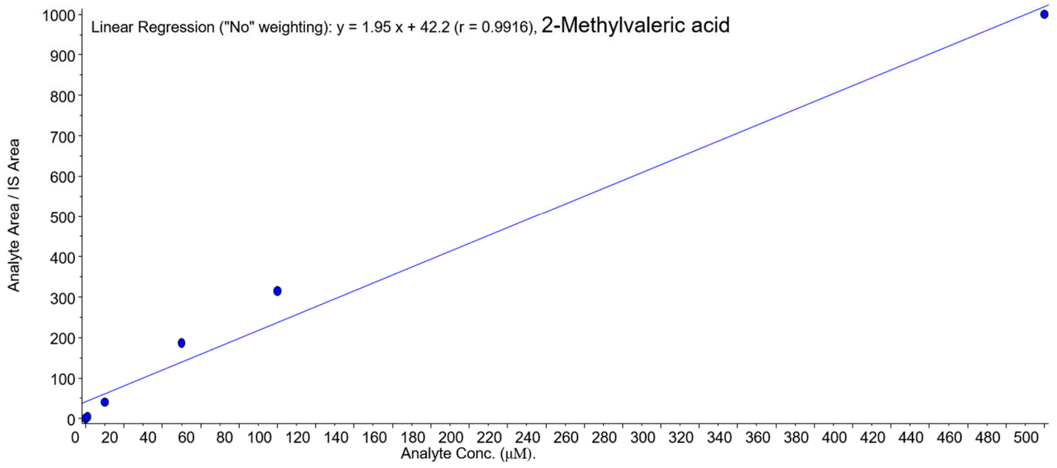
Linear Regression ("No" weighting): y = 1.95 x + 42.2 (r = 0.9916), 2-Methylvaleric acid
Analyte Area / IS Area
Analyte Conc. (µM).

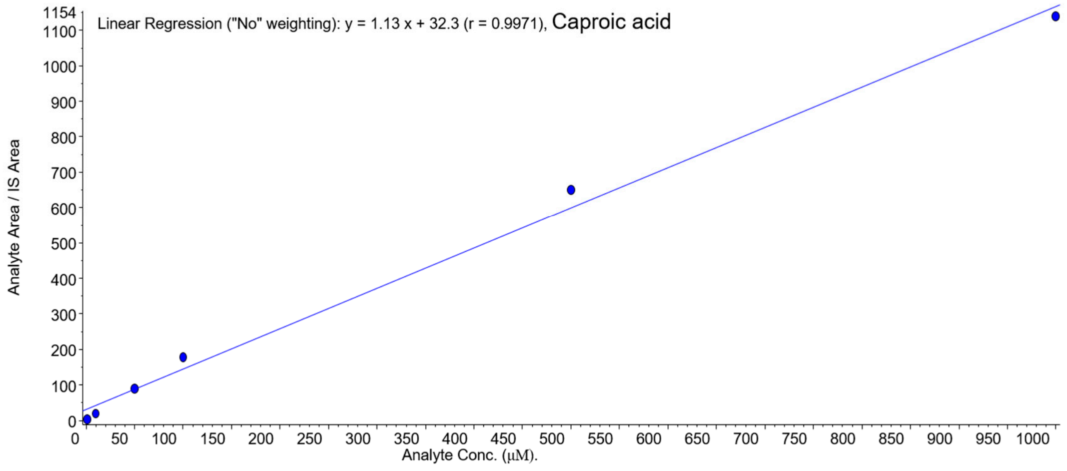
Linear Regression ("No" weighting): y = 1.13 x + 32.3 (r = 0.9971), Caproic acid
Analyte Area / IS Area
Analyte Conc. (µM).

Figure S3. Calibration curves for 12 SCFAs. Calibration range for acetic acid: 1 μM - 1 mM; 2,2-dimethypropionic acid, isovaleric acid, 2,2-dimethylbutyric acid, and 2-ethylbutyric acid: 100 nM - 100 μM; 2-methybutyric acid and 2-methylvaleric acid: 100 nM - 500 μM; Other SCFAs: 100 nM - 1 mM.

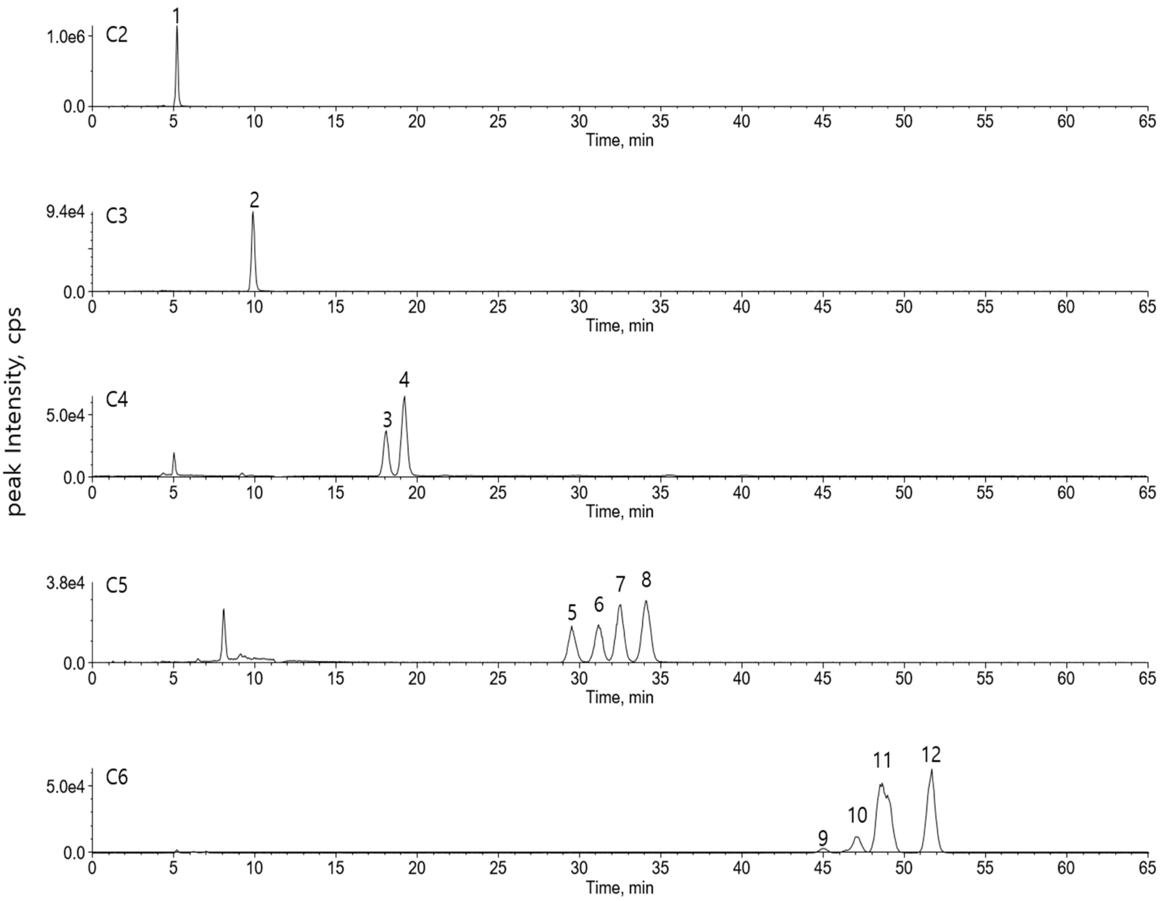

Figure S4. Extracted ion chromatograms of 12 SCFAs in a standard solution (10 μM). The number on top of each peak represents each SCFA (1: acetic acid; 2: propionic acid; 3: isobutyric acid; 4: butyric acid; 5: 2,2-dimethylpropionic acid; 6: 2-methylbutyric acid; 7: isovaleric acid; 8: valeric acid; 9: 2,2-dimethylbutyric acid; 10: 2-ethylbutyric acid; 11: 2-methylvaleric acid; 12: caproic acid). IS represents internal standard. LC separation was performed at a flow rate of 400 μL/min and a temperature of 23 °C. The separation gradient was as follows: 30 % B at 0 min, 30 to 40 % B in 60 min, 40 to 30 % B in 0.1 min, and 30 % B in 4.9 min. The chromatograms were obtained using the same experimental conditions described in Materials and Method, except LC flow rate, column temperature, and gradient.
